# In the Long Run: Physical Activity in Early Life and Cognitive Aging

**DOI:** 10.3389/fnins.2019.00884

**Published:** 2019-08-27

**Authors:** Charlotte Greene, Hyunah Lee, Sandrine Thuret

**Affiliations:** ^1^GKT School of Medical Education, King’s College London, London, United Kingdom; ^2^Department of Basic and Clinical Neuroscience, Institute of Psychiatry, Psychology & Neuroscience, King’s College London, London, United Kingdom

**Keywords:** cognition, cognitive decline, exercise, early life, old age, cognitive reserve, prevention

## Abstract

A certain degree of age-related cognitive decline is normal; however, some people retain more cognitive function than others. Cognitive impairment is associated with an increased risk of dementia. Thus, understanding the factors that contribute to cognitive reserve is crucial, so effective strategies for the prevention of dementia can be developed. Engaging in physical activity can delay cognitive decline and reduce the risk of dementia and a number of early life conditions have been shown to have long-lasting effects on cognition. This mini-review combines these two observations to evaluate the evidence from both animal models and epidemiological studies for physical activity in early life (≤30 years) delaying cognitive decline in later life (cognition tested ≥60 years). Three epidemiological studies were found; two showed a positive association and one found none. The latter was deemed to have an unreliable method. A review of animal studies found none that analyzed the effect of physical activity in early life on cognition in later life. However, in rodent models that analyzed mid-life cognition, runners showed improved cognition and enhanced adult hippocampal neurogenesis, changes which were preserved across the life span. Currently, there is insufficient evidence to conclude whether physical activity in early life may delay cognitive decline in later life, but these results indicate that further studies are warranted. Future human research should be in the form of longitudinal studies that begin below ≤15 years and assess sex differences. Crucially, the physical activity data must define type, quantity and intensity of exercise.

## Introduction

The aging brain exhibits widespread detrimental changes in structure, including reduction in size, plasticity and cerebral blood flow, and also in function (Peters, 2006). While some age-related cognitive decline in memory (particularly semantic memory), executive function, and processing speed may be considered normal (Harada et al., 2013), more significant decline in these than expected for a person’s age can be classified as either cognitive impairment with no dementia (CIND) or mild cognitive impairment (MCI), depending on which cognitive domains are most affected (Blondell et al., 2014). A diagnosis of CIND or MCI has been associated with increased risk of dementia (Blondell et al., 2014). A large-scale, longitudinal study of individuals aged ≥55, showed that those with MCI were 3.89 times more likely to be diagnosed with dementia later in life than those without (de Bruijn et al., 2014).

Dementia is a devastating condition not only for the individual and their loved ones, but also poses an enormous and growing social and economic burden for public health. Currently, over 5% of the aged ≥65 population suffer from dementia and by 2050 it has been estimated that 22% of the entire population will be aged ≥60, equating to approximately two billion people of that age group (Blondell et al., 2014; Shatenstein et al., 2015). To reduce the incidence of dementia, delay the age of onset, and improve quality of life for this subpopulation, we need to understand what factors can contribute to slowing age-related cognitive decline.

Evidence suggests engaging in physical activity may delay cognitive decline and reduce the risk of dementia and a number of early life conditions have been shown to have long-lasting effects on cognition. In this review, we combine these two observations and evaluate the existing evidence from epidemiological studies for physical activity in early life (≤30 years) delaying cognitive decline in later life (≥60 years) and from rat models on the effect of physical activity in early life (≤4 months) on cognition in later life (≥7 months). First, we explore the idea of cognitive reserve, and the significance of early life events on its development. Next, we discuss why physical activity is an important factor to consider. We then review the data generated from epidemiological studies and animal models that met our inclusion criteria and search terms.

## Early Life Conditions and Cognitive Reserve

The brain is capable of change and adaptation as a result of experience, termed plasticity. Previous studies have suggested there is no direct relationship between brain pathology and cognitive impairment (Walhovd et al., 2016). Alzheimer’s disease lesions are commonly found upon post-mortem in people who were cognitively intact before death, termed asymptomatic AD (Iacono et al., 2009). Likewise, individuals with similar levels of brain pathology can show different levels of cognitive impairment (Fritsch et al., 2007). These observations have led to the rise of the term “cognitive reserve” – the idea that people can build up a reserve of neurological and/or cognitive resources that increase the tolerance toward damaging stimuli. Various studies have shown factors that might serve as the biological basis of cognitive reserve including greater cortical surface area and neuronal hypertrophy (Iacono et al., 2009; Walhovd et al., 2016). Hypertrophy of the cell bodies (+44.9%), nuclei (+59.7%), and nucleoli (+80.2%) of neurons in the CA1 region of the hippocampus was found in the brains of those with asymptomatic AD compared with those with MCI, suggesting that this may be one of the mechanisms that protects against cognitive decline (Iacono et al., 2009).

Recently, there has been increasing interest into whether early life conditions can affect this reserve. Lower birth weight, body length and head circumference, have been shown to correlate with faster cognitive decline and increased incidence of dementia rates (Raikkonen et al., 2013). Childhood nutritional status (Zhang et al., 2010), linguistic ability (Calvo et al., 2016), stress and adversity (Radford et al., 2017), and education (Zahodne et al., 2015) have also been linked to cognition in later life.

## Physical Activity and Cognitive Function

Evidence from both epidemiological studies and randomized control trials suggests that engaging in physical activity improves cognitive function at various stages of life, from early (Sibley and Etnier, 2003; Scudder et al., 2014) to mid and late life (Yaffe et al., 2001; Jedrziewski et al., 2010; Nishiguchi et al., 2015). In a meta-analysis of 47 longitudinal studies with participants aged ≥40 years, Blondell et al. (2014) showed that people with higher levels of physical activity had a lower risk of progressive cognitive decline (RR 0.65, 95% CI 0.55–0.76) and dementia (RR 0.86, 95% CI 0.76–0.97).

Contradictory evidence was presented by Sabia et al. (2017) who found no neuroprotective effects of mid-life physical activity and suggested that previous findings were attributable to reverse-causation (people in the pre-clinical dementia phase exercise less). Their follow-up time of 28 years is the longest of the studies discussed. However, the study with the longest follow-up time, 26 years, in the review by Blondell et al. (2014) found a significant association between physical activity and cognition. The longer-term impact of physical activity across the life-span merits more attention. Indeed, in their sample of 226 middle aged and older adults (≥55 years), Gill et al. (2015) observed that better global cognitive performance was associated with higher lifetime physical activity (*p* = 0.045), physical activity between 0–20 years (*p* = 0.036) and 21–35 years (*p* < 0.0001).

Numerous animal studies have shown positive correlations between exercise and cognition and proposed several possible neurobiological mechanisms for the enhancement of cognitive reserve – adult hippocampal neurogenesis and suppressed apoptosis, increased production of insulin-like growth factor and brain-derived neurotrophic factor (BDNF) and modulated cytokine signaling (Kim et al., 2010; Cassilhas et al., 2012; Gomes da Silva et al., 2012; Speisman et al., 2013). Enhanced neurogenesis in the dentate gyrus (DG) of the hippocampus has received particular attention. Neurogenesis in the mammalian DG declines with age, while an increase leads to improved cognitive function (Kempermann, 2015). Exercise in rodents can induce a 2–3 times increase of newly generated hippocampal neurons, an effect detected as early as 24 h on, and improve neuronal complexity by boosting dendritic spine density (Saraulli et al., 2017).

Research into whether different levels of exercise intensity produce varying effects on cognition has yielded inconsistent results. In rats, running at a speed of 25 m/min for 12 weeks (the severe exercise group) led to shrunken hippocampal neurons with damaged mitochondria (Sumitani et al., 2002) and only low-intensity running (15 m/min), not moderate (25 m/min), increased the expression of BDNF in the hippocampus (Soya et al., 2007). Indeed, at moderate-intensity running, induction of BDNF mRNA was depressed (Soya et al., 2007). Inoue et al. (2015a, b) more precisely defined the intensity of exercise by training rats below (aerobic) and above (anaerobic) the lactate threshold, discovering that adult hippocampal neurogenesis was increased only by aerobic exercise. However, in their study Lee et al. (2018) found that both intense and moderate exercise prevented cognitive decline from chronic stress and improved newborn cell survival and blood vessel density. Moderate exercise sessions involved running at 20 m/min for 60 min, while during intense sessions the treadmill speed was increased from 30 m/min until the mouse became exhausted (most at 50 m/min). The blood lactate concentration exceeded 14 mmol/L immediately after running, suggesting that the lactate threshold was exceeded.

The results of human studies are also mixed. One study reported that individuals who received aerobic training showed substantial improvements in cognitive function compared to those on the anaerobic program (Kramer et al., 1999). In a study of 36 female college students the high-intensity resistance (primarily anaerobic) group (resistance exercises; 28 repetitions, 80% 1RM) showed a worse cognitive performance compared to those on a moderate-intensity mixed program (resistance exercises; 12 repetitions, 30% 1RM, aerobic; 30 min walking) and controls (Chang et al., 2017). While, a meta-analysis of 17 studies found that both aerobic and resistance exercises were effective in improving cognition in adults with MCI (Wang et al., 2019).

Brown et al. (2012) measured routine levels of physical activity via actigraphy in 217 participants aged 60–89 years and reported a significant association between intensity, but not volume, of physical activity and cognitive functioning. Analysis was done by stratifying the cohort into tertiles based on physical activity intensity. Given this method and the age of participants it seems unlikely that those in the highest tertile of physical activity intensity reached the intensity of the training of other studies (e.g., Chang et al., 2017). The conflicting results in different studies may well be partially explained by the difficulty in defining “high intensity” and the heterogeneity of classifications used. The question of what level of intensity, and type (aerobic or resistance/anaerobic) may be most beneficial for cognitive function and enhancing cognitive reserve needs to be researched further.

## Epidemiological Studies on the Effects of Early Physical Activity on Late Life Cognition

Children’s brains are uniquely malleable, but the brain continues to develop into the third decade of life (Johnson et al., 2009; Petanjek et al., 2011). Thus, early life will be defined as ≤30 years. An adequate assessment of cognitive aging is defined as cognitive tests completed ≥60 years, when structural age-related changes to the brain would have begun (Nyberg et al., 2012). A further inclusion criterion was the use of statistical adjustment, including adjusting for exercise at other times in life, to ensure it was specifically exercise in the early period that was analyzed. Exclusion criteria were not in English, a conference paper and published later than 2018. The following search terms – ((((early life OR adolescence OR early adulthood)) AND (physical activity OR exercise)) AND (cognitive aging OR cognit^∗^)) AND (old age OR late) – were applied on PubMed and Embase. Three studies met the inclusion criteria.

As shown in Table 1, two studies showed a positive association between early life exercise and cognitive function later in life (Dik et al., 2003; Middleton et al., 2010), and one study showed no association (Fritsch et al., 2007). None looked at the effect of exercising ≤15 years of age, an obvious immediate gap in research.

**TABLE 1:** Characteristics and results of studies investigating the correlation between early life (≤30) physical exercise and cognitive aging (≥60).

**Study/country**	**Participants**	**Physical activity**	**Cognitive tests**	**Statistical adjustment**	**Results after full adjustment**
Dik et al., 2003/Netherlands	1,241 – M (48.7%) and F (51.3%), aged 62–85 (mean 74.9), CI excluded, No info on ethnicity	15–25 years Asked retrospectively no. of hours per week	GCF (MMSE), SP (Alphabet coding task-15)	Confounders: age, sex, verbal intelligence, SES, lifestyle (early life physical work, current physical activity, smoking, alcohol), health indicators (diabetes, cardiac disease, depression)	GCF – no significance, SP – M only positive association for low (beta = 0.97) and mod (0.67) high – insignificant negative association (−1.04)
Fritsch et al., 2007/United States	349 – M (42.4%) and F (57.6%), aged 74 – 76 (mean 74.8), CI excluded, 99.7% white	16–18 years Information collected from yearbooks – grouped according to number of physical activities	GCF (TICS-m), SP (Timed months of the year backward test), episodic memory (Logical memory A subtest of the wechsler memory scale), verbal fluency (animal naming)	Path analysis for: sex, teen IQ, parent’s SES, HS physical, mental, social activities, ML mental, physical and social occupational demands, education	No association
Middleton et al., 2010/United States	9344 – only F, ≥65 years (mean 71.6), “primarily white” – no figures provided	“teenage” Asked retrospectively about low, mod, high intensity exercise – modified Paffenbarger questionnaire	GCF (mMMSE)	Confounders: age, education, marital status, diabetes, hypertension, depressive symptoms, smoking, BMI	Physically active lower prevalence of CI vs. inactive – 8.5 vs. 16.7% 0.65 OR (0.53–0.80)

*M, men; F, female; CI, individuals with cognitive impairment; GCF, global/general cognitive functioning; SP, speed processing; HS, high school; ML, midlife.*

Fritsch et al. (2007) drew participants from the population of a longitudinal aging study of students who graduated from high school 1945–1947. The authors assessed the extent of students’ physical exercise while attending the school aged 16–18 by gathering information from their yearbooks. They defined physical activities as “athletic clubs including sports teams, dance clubs, honor groups associated with athletic performance, and cheerleading” and grouped the participants based on the number of activities mentioned in the yearbook. Although this data is free from recall error and bias it has significant weaknesses; it covers the short period of 2 years and only exercise in school. Most importantly, the analysis is based on the number of activities rather than the time spent engaged in them. Someone who excels at one sport may well be a member of only that team. They could devote significant time to it, but they would be considered by this study to have a low level of physical activity. This method also does not include analysis of the intensity of physical activity, an important omission given the evidence discussed above that it may be specifically mild, aerobic forms that are beneficial. Thus, the conclusion of no observable association is unconvincing and can be ignored unless the results are replicated in other independent studies.

The two studies that showed a positive association had substantially larger sample sizes (1241 and 9334 vs. 349). Dik et al. (2003) found an improvement in processing speed, but not global/general cognitive functioning (GCF), only in men in the low (≤1–2 h per week) and moderate exercise (3–9 h per week) groups. The authors provide a convincing explanation for why this trend was not seen in the high-level exercise cohort (≥10 h per week). Fifty-six percent of those men had a high level of physical work. Not only may manual labor be associated with poor working conditions and exposure to harmful substances, it is likely to be a static and anaerobic form of exercise. Indeed, separate analysis for sport and work-related activities revealed the negative association of the high exercise men could be attributed to physical work. In contrast, Middleton et al. (2010) observed a positive association in 9344 women. Teenage physical activity was correlated with lower odds of late-life cognitive impairment (defined as an mMMSE 1.5 SD ≤ mean) compared to physical inactivity – 8.5 vs. 16.5%. Four age categories were included in the study (teenage, 30, 50, and late life) and when a separate comparative analysis was carried out teenage physical activity was most strongly associated, OR = 0.73 (0.58–0.92).

In their assessment of GCF all three studies used a version of the Mini-Mental State Examination (MMSE; Folstein et al., 1975). Dik et al. (2003) used the MMSE itself, Middleton et al. (2010) the modified MMSE (mMMSE), and Fritsch et al. (2007) the Modified Telephone Interview for Cognitive Status (TICSm; Welsh et al., 1993). The mMMSE is a shortened version which evaluates orientation, concentration, memory, and praxis but omits questions regarding language. The TICSm was developed as a telephone version of the MMSE and correlates highly with it (*r* = 0.8) (Fritsch et al., 2007). It includes orientation, concentration, memory, naming, comprehension, and abstraction. The MMSE is a widely used and respected tool designed as a screening test for cognitive impairment with a maximum score of 30, and normal cognitive function usually set at a score ≥24 (Creavin et al., 2016). A recent Cochrane review (Creavin et al., 2016) with the objective of determining the diagnostic accuracy of the MMSE for dementia in patients ≥65 found that the summary accuracy at a cut point of 24 was sensitivity 0.85 and specificity 0.90. For speed processing Dik et al. (2003) employed the Alphabet Coding Task-15 (Piccinin and Rabbitt, 1999) while Fritsch et al. (2007) used the Timed Months of the Year Backward Test (Ball et al., 1999), both are validated timed tests for the assessment of central speed processing. The fact different cognitive tests were used is a limitation when comparing the results of the studies. However, there are only minor differences between the assessments used and the MMSE as an effective measure of cognition is supported by a strong evidence base.

A limitation of two of the three studies is that the data provided about exercise is retrospective and self-reported by participants. Asking people, some of whom may have cognitive impairment, to report on their exercise levels of decades before, can be problematic, making defining the quantity, intensity and type of exercise difficult. A shared weakness of all three is that the majority of participants were white. Most importantly, isolating the impact of physical exercise in early life given all the confounding variables throughout life is challenging.

Given only three studies could be found that fit our inclusion criteria, there is insufficient evidence to draw a conclusion. However, the results suggest that further research is warranted. Animal models can remove confounding variables more effectively than human studies and may also provide evidence of a mechanism.

## Animal Studies on the Effects of Early Physical Activity on Late Life Cognition

The following inclusion criteria were used for rat models; a period of exercise ≤4 months of age, no further exercise after this, and neurogenesis or cognition tested ≥7 months of age. Exclusion criteria were not in English, a conference paper and published after 2018. In an initial search no animal models were found that analyze the effect of physical activity in early life on cognition in later life and thus the age limit was reduced to 7 months (corresponding to mid-life). The impact on mid-life cognition is relevant and worthy of discussion. Evidence of a neurobiological mechanism that can explain how exercise may exert a long-term effect on cognition, supports the idea that there should be future research into this subject.

Our criteria and the following search terms – ((((early life or early age)) AND (running or exercise)) AND (neurogenesis OR learning or cognt^∗^)) AND (adult or aging or old age) – were applied on PubMed and Embase and yielded two results.

Shevtsova et al. (2017) divided one-month-old male rats into two groups, control (*n* = 40) and runners (*n* = 40) that had free access to a running wheel for 6 weeks. After 4 months, the rats were trained on a contextual fear (CF) conditioning task, the learning of which is known to depend on newborn hippocampal neurons. Two weeks later (aged 7 months) they tested for memory of the CF response in the same, similar or different environment. Runners performed better and froze less in similar and different conditions than non-runners, showing that their memory was enhanced. Interestingly, all rats learned the CF task at the same rate. It was specifically the runners’ memory that improved (Shevtsova et al., 2017).

The study’s second objective was to compare neuronal activity in adult-born and developmentally born neurons in the DG. Three weeks before CF conditioning, rats were injected with chloro-deoxyuridine (CldU), a thymidine analog, to mark a proportion of 5-week-old neurons at the time of testing. To see the activity of these neurons, an immediate early gene (c-Fos) was labeled, and the densities of double-labeled cells (c-Fos/CldU) were measured. The results were that these adult-born neurons were more active than developmentally born neurons, with a higher percentage active in early runners compared to controls. This is evidence for two significant points; that adult neurogenesis is important for learning and memory and that it is enhanced in the long term by physical exercise early in life.

Merkley et al. (2014) divided 4-week-old male rats into controls (*n* = 24) and runners (*n* = 28) that had access to a running wheel for 30 days. Both cohorts were subdivided into four groups based on the time between cessation of running and perfusion. They were perfused 1 week, 5 weeks, 6 months, and 9 months after the removal of the wheel. Various immunohistochemistry labels were used to mark stages of neuronal development in the DG. The results showed significant age-related declines in the number of all types of cells in both cohorts indicating that neurogenesis decreases with age. 5 weeks post-running rats (14-week-old) had significantly more adult-born neurons (4-week-old) that had survived and matured than controls (2300 vs. 1500 mean number of cells per DG). The rate of decay of these neurons with age was almost identical in both cohorts. Therefore, exercise-induced changes were preserved across the life span. This is further evidence that early life exercise has long term effects on DG neurodevelopment.

A limitation of both studies is that only male rats were tested. This is particularly relevant given the sex-dependent trends in the epidemiological data and the results of a rodent study that found running enhanced neurogenesis solely in males (Saraulli et al., 2017).

Rats were tested at 7–11 months of age, which as discussed above corresponds to mid-life rather than late-life. Only after further studies have tested later in life (e.g., ≥15 months when reproductive senescence begins) can findings be considered comparable to the effect of physical activity in early life on age-related cognitive decline in humans. The results of these two studies of mid-life cognition suggest that such models should be carried out.

## Conclusion

The paucity in both human and animal studies is clear. Of the three epidemiological studies that analyzed early life exercise ≤30 years and cognitive function ≥60 years, two found a positive association and one found none. While the latter (Fritsch et al., 2007) seems to have used a relatively unreliable method for assessing the amount of physical activity, the two larger-scale studies did find a positive association. However, Dik et al. (2003) observed a positive association only in processing speed, not in GCF, and only in men in the low and moderate exercise groups, whereas Middleton et al. (2010) found association in their cohort of women and for GCF.

A review of animal studies found none that analyzed the effect of physical activity in early life on cognition in later life. However, in rat models that analyzed mid-life (≥7 months) cognition, runners showed improved cognition and enhanced adult hippocampal neurogenesis, changes which were preserved across the life span. This provides a possible neurobiological mechanism for how early life exercise may exert long term effects on cognition.

There is insufficient evidence to conclude physical activity in early life delays cognitive decline in later life. However, the results from both epidemiological and animal studies indicate that further studies are warranted. Future human research should be in the form of longitudinal studies that address the lack of data on physical activity ≤15 years and the conflicting results for men and women by performing sex stratified analysis. Crucially, the physical activity data must define type, quantity and intensity of exercise. The results of Dik et al. (2003) and a significant body of evidence discussed above suggests that the benefit of physical activity may vary with intensity and type (aerobic or anaerobic/resistance).

## Author Contributions

CG carried out the study and wrote the manuscript. HL reviewed and contributed to the writing of the manuscript. ST conceived and reviewed the manuscript.

## Conflict of Interest Statement

The authors declare that the research was conducted in the absence of any commercial or financial relationships that could be construed as a potential conflict of interest.
